# Bowel obstruction caused by colonic metastasis of lung adenocarcinoma: a case report and literature review

**DOI:** 10.1186/s12957-019-1611-y

**Published:** 2019-04-08

**Authors:** N. A. Parker, C. McBride, J. Forge, D. Lalich

**Affiliations:** 1 Department of Internal Medicine, University of Kansas School of Medicine, 2817 N Tallgrass St, Wichita, KS 67226 USA; 2 Department of Internal Medicine, University of Kansas School of Medicine, 1010 N Kansas St, Wichita, KS 67214 USA; 3 Department of Anatomical and Clinical Pathology, Wesley Medical Center, 550 N. Hillside St, Wichita, KS 67214 USA

**Keywords:** Colonic metastasis, Primary lung cancer, Non-small cell lung cancer

## Abstract

**Introduction:**

Lung cancer is the most common cause of cancer-related deaths globally. Metastatic disease is often found at the time of initial diagnosis in the majority of lung cancer patients. However, colonic metastases are rare. This report describes an uncommon case of colonic metastasis from lung adenocarcinoma.

**Case presentation:**

A 64-year-old female presented to her gastroenterologist for progressively worsening abdominal pain and constipation. Exploratory colonoscopy revealed a large rectosigmoid mass resulting in near total rectal occlusion. Her specialist recommended she immediately go to her regional hospital for further workup. On admission, she complained of continued abdominal pain and constipation. Notably, she had a past medical history of non-small cell lung cancer (T1bN3M0 stage IIIB), diagnosed 1 year prior. She was thought to be in remission following radiation and immunotherapy with pembrolizumab. Upon hospital admission, she underwent an urgent colostomy, ileocecectomy and anastomosis, and rectosigmoid mass resection with tissue sampling. Pathology confirmed the diagnosis of colonic metastasis from primary lung adenocarcinoma. Treatment was with systemic chemotherapy followed by localized radiation to the pelvic region was started. She did not respond well to these therapies. Subsequent imaging showed refractory tumor growth in the pelvic region. Treatment could not be completed due to the patient experiencing a debilitating stroke, and she was transitioned to hospice care.

**Conclusions:**

Clinicians should have a low threshold for intestinal investigation and considerations for colonic metastasis when patients with a history of primary lung cancer have abdominal symptoms.

## Background

Non-small cell lung carcinoma (NSCLC) accounts for the majority of all lung cancer cases. Metastatic disease is often present at the time of diagnosis, regardless of primary lung cancer type [1–4]. However, colonic metastases are rare. The exact prevalence of large bowel metastasis is difficult to determine. Asymptomatic colonic metastasis has an incidence of approximately 12% based on autopsy studies [5–10]. Symptomatic colonic metastasis infrequently occurs [5, 6, 8, 11–14]. Clinicians should have a high index of suspicion and a low threshold for intestinal tract investigation when primary lung cancer patients present with abdominal symptoms.

## Case report

**Fig. 1:**
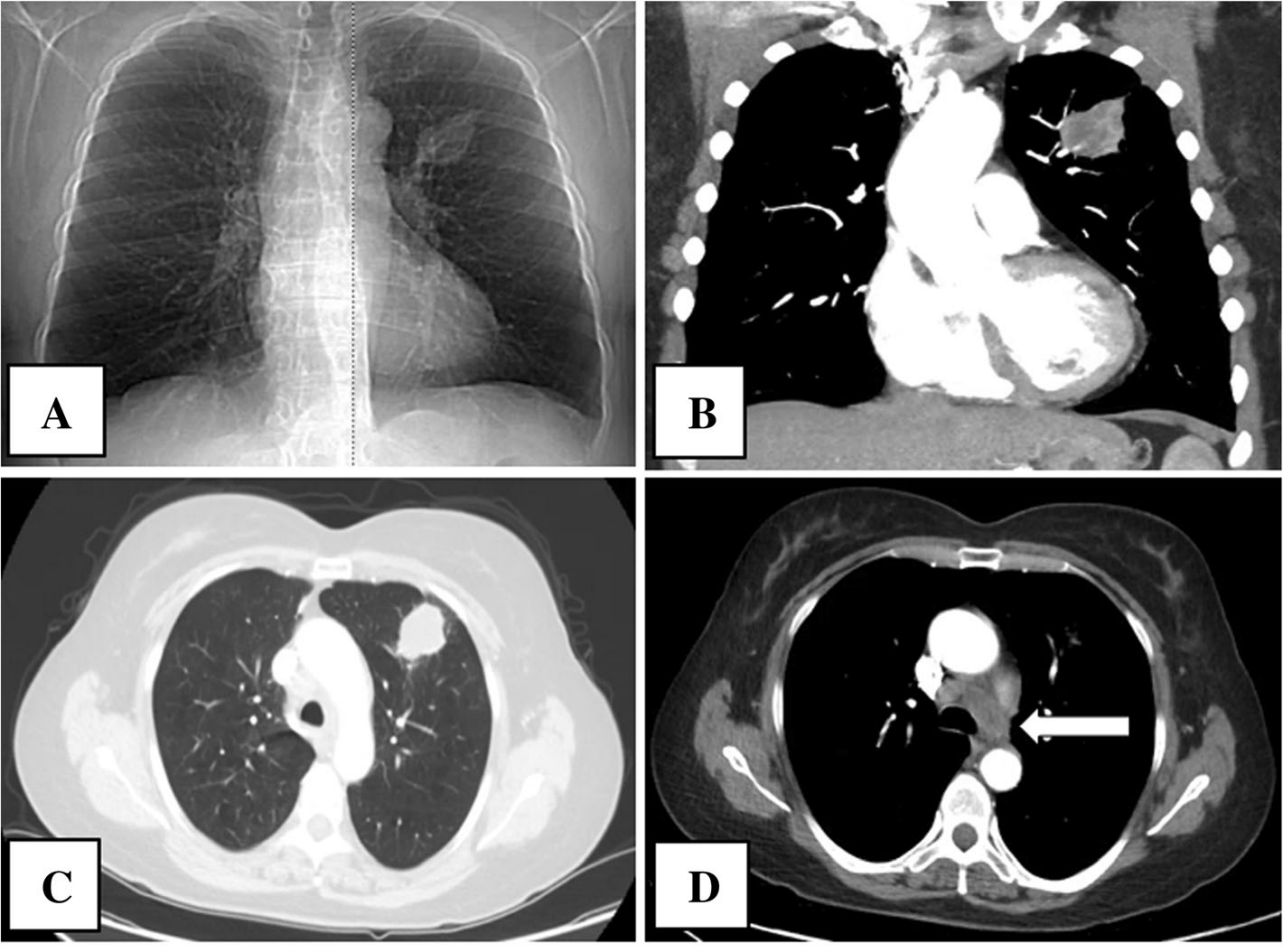
Chest X-ray and computed tomography showed a tumor in the left lung field. **a** CXR showed a round mass in the left upper lung field. **b** CT coronal image demonstrated the mass anteriorly within the left upper lobe. **c** CT scan revealed a 3.4 × 3.1 cm left upper lobe pulmonary mass lesion most compatible with primary lung cancer. **d** CT scan showed abnormal left hilar and mediastinal adenopathy (*arrow*) suggestive of metastatic nodal involvement

A 64-year-old female was referred to the hospital by her gastroenterologist after a same-day colonoscopy revealed a large rectosigmoid mass resulting in near total rectal occlusion. She had a past medical history of tobacco smoking and NSCLC (T1bN3M0 stage IIIB), diagnosed 1 year prior (Fig. 1). She was thought to be in remission following radiation and immunotherapy with pembrolizumab.

**Fig. 2:**
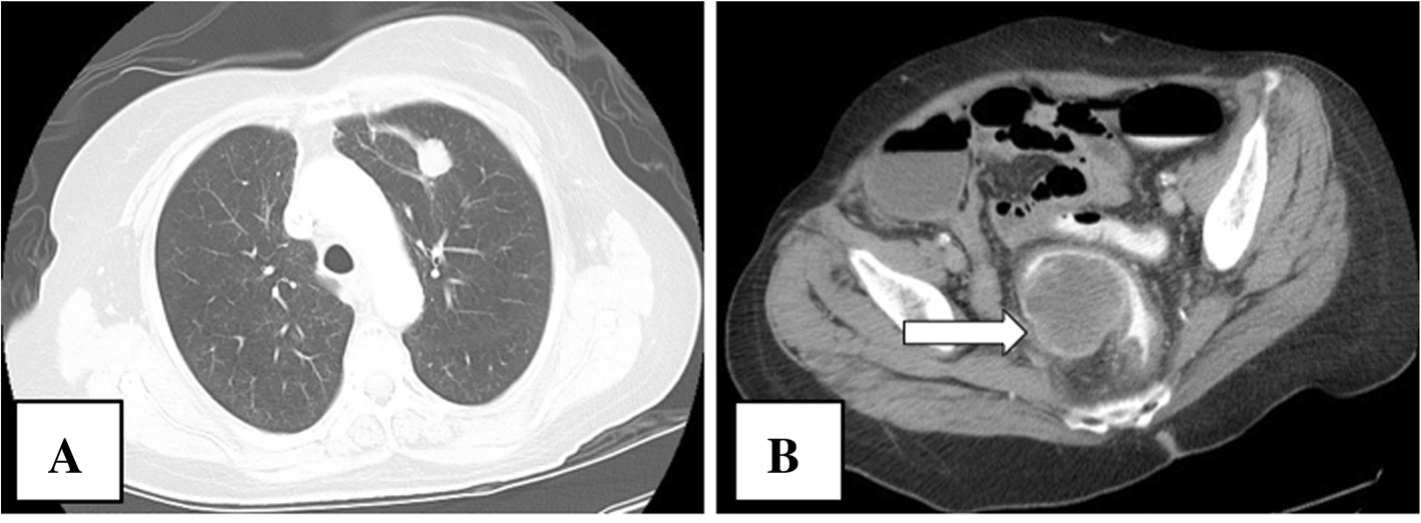
Imaging showed a tumor in the left lung field and sigmoid colon. **a** Chest CT revealed a residual soft tissue mass anteriorly within the left upper lobe measuring approximately 2.0 × 1.7 cm without appreciable adenopathy (not shown) consistent with the patient’s known history of lung cancer. **b** Abdomen and pelvic CT showed a soft tissue mass with approximate 5.0 × 4.7 cm dimensions within sigmoid colon (*arrow*) at 15 cm from the anal orifice. A sigmoid mass with extrinsic features and mucosal involvement can be seen contributing to marked narrowing of the sigmoid colon, but allowed contrast to pass through area of narrowing

On admission, she complained of progressively worsening abdominal pain and constipation. Vital signs and measurements were unremarkable. Physical examination was primarily benign. Notable laboratory findings only included elevated carcinoembryonic antigen of 4.2 ng/dL. Computerized tomography (CT) imaging showed a severe colonic stool burden and a soft tissue left upper lobe lung mass consistent with patient’s NSCLC history. A single large soft tissue mass with possible mucosal invasion in the rectosigmoid colon was noted (Fig. 2). She underwent urgent diagnostic laparoscopy that was quickly converted to open exploratory laparotomy due to numerous bowel-to-bowel and bowel-to-anterior abdominal wall adhesions. At that time, a rectal mass appeared to be invading into the small bowel. Ultimately, colostomy, ileocecectomy and anastomosis, and rectosigmoid mass resection with tissue sampling were performed. She tolerated the procedure well, and her immediate postoperative course was uneventful.

**Fig. 3:**
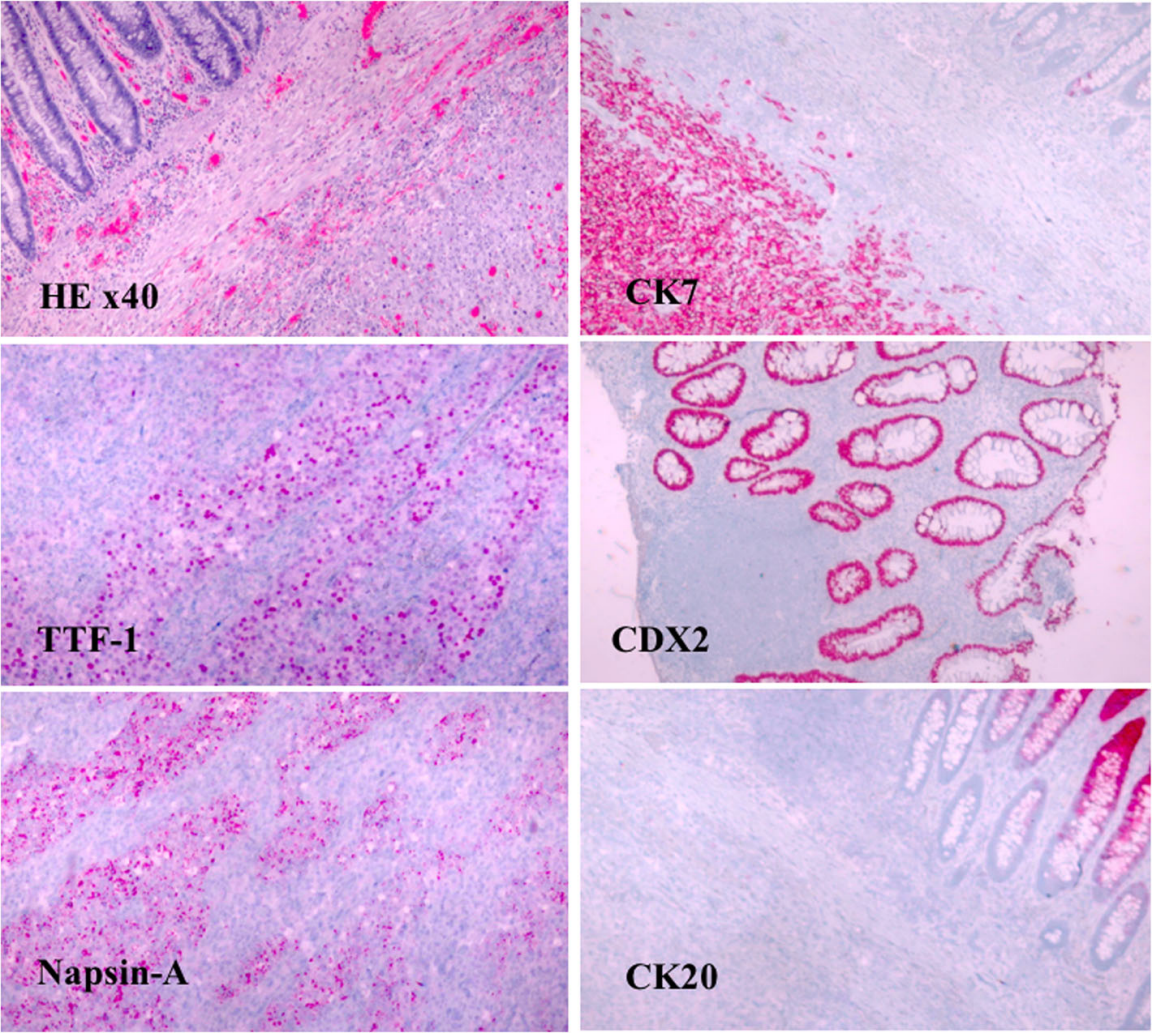
The pathology specimen demonstrated metastatic lung adenocarcinoma of the colon. (H&E stain, × 40). The carcinoma cells were positive for CK7, TTF-1, and Napsin-A, but negative CK20 and CDX2 (× 40)

Rectosigmoid mass biopsies revealed positivity for high-grade NSCLC and favored metastatic poorly differentiated adenocarcinoma of lung origin. Hematoxylin and eosin (H&E) staining showed rectosigmoid mass tissue exhibiting extensive necrosis, focal mucosal involvement, and negativity for regional lymph node carcinoma. Also, normal appearing colonic glandular cells were surrounded by atypical cells infiltrating the colonic stroma. To evaluate these high-grade and poorly differentiated malignant changes further, properly controlled routine immunohistochemical (IHC) stains for cytokeratin 7 (CK7), thyroid transcription factor-1 (TTF-1), Napsin-A, epithelial specific antigen/EpCAM (Moc-31), Ber-EP4, p63, cytokeratin 5 or 6 (CK5, CK6), caudal type homeobox 2 (CDX2), and cytokeratin 20 (CK20) were performed not only based on the patient’s age, gender, and past medical history, but also her recent clinical, radiologic, and operative findings. Additional properly controlled IHC stains for leukocyte common antigen (CD45), melanoma antigen recognized by T cells (MART-1), gross cystic disease fluid protein 15 (GCDFP-15), estrogen receptor (ER), synaptophysin, neural-cell adhesion molecule (NCAM/CD56), and chromogranin were performed due to the unusual presentation and nature of the case. The malignant cells exhibited strong positive immunoreactivity for CK7, and positive TTF-1 Napsin-A, Moc-31, and Ber-EP4, while showing only minimal focal staining for p63 and cytokeratin 5 or 6 (CK5, CK6). The tumor was negative for CDX2, CK20, CD45, MART-1, GCDFP-15, ER, synaptophysin, NCAM/CD56, and chromogranin (Fig. 3). Mucicarmine staining was equivocal for intra-cytoplasmic mucin. This IHC staining profile (strongly positive CK7 and positive TTF-1/Napsin-A with negative CDX2/CK20) supported metastatic adenocarcinoma of lung origin, rather than primary colorectal adenocarcinoma. This hypothesis was supported by numerous colonic and regional lymph node samples lacking malignant carcinoma cells and properly controlled IHC stains of right colon and ileum biopsy cells exhibiting negative immunoreactivity for CK7, TTF-1 Napsin-A, Moc-31, and Ber-EP4.

Her postoperative course was uneventful, and she was discharged home. The patient was started on systemic chemotherapy with carboplatin and pemetrexed followed by radiation to the pelvic region for metastatic NSCLC. She did not tolerate chemoradiation therapy well. During the treatment period, she developed considerable pelvic pain resulting in a significant performance status decline. She also experienced multiple prolonged hospitalizations due to infections. Subsequent positron emission tomography–CT (PET-CT) scans suggested refractory pelvic tumor growth. Additional radiation for palliation of pain by reducing pelvic tumor size was determined reasonable. However, the patient experienced a debilitating stroke and was transitioned to hospice care.

## Discussion

The most common NSCLC metastatic site is bone (34%), followed by lungs (32%), brain (28%), adrenal glands (17%), liver (13%), and extrathoracic lymph nodes (9%) [15]. Colonic metastasis is uncommon with an incidence of 0.1% [16]. Although metastasis to the colon from lung cancer is uncommon, the phenomenon has been reported [5–56]. Most commonly, the small intestine develops metastatic lesions [5]. This could be due to the enhanced potential of small bowel malignancies to cause serious complications such as perforation, obstruction, or bleeding [5, 6]. Only 44 unique case reports of lung cancer metastasizing to the colon have been published globally (Table 1) [5, 10, 13, 17–56]. The pathological diagnosis in 20 of the 44 cases (45%) was squamous cell carcinoma (SqCC) [13, 17–35]. Twelve lung adenocarcinomas (27%) and five small cell lung carcinomas (SCC, 11%) were confirmed as primary origins [5, 10, 36–44, 46–49, 52]. Large-cell carcinoma of the lung was reported in three cases (7%) [10, 50, 51]. Four cases (9%) confirmed colonic metastasis from other primary lung histopathologic cell types such as sarcomatoid, pleomorphic, and unknown [53–56]. SqCC had a higher propensity for colonic metastasis [13, 17–35]. Lung adenocarcinoma had the second highest potential for colonic metastasis [10, 36–44, 52]. Abdominal pain due to intestinal tract obstruction was the most frequent initial clinical symptom of metastatic colon cancer from primary malignant lung neoplasms [19, 25, 29, 32, 33, 37, 38, 40, 43, 44, 52, 53]. Bloody stool due to either melena or hematochezia was also a common chief complaint [5, 10, 17, 31, 35, 48, 56]. Diagnosis of metastatic lung cancer to the colon by incidental polyp discovery occurs infrequently [10, 18, 42, 55]. Metastatic colonic neoplasms of lung origin can also present initially with non-bloody diarrhea, encopresis, and hyponatremia [20, 36, 39]. Metastatic lung SqCC and SCC to the colon were associated more with serious complications such as perforation, hemorrhage, and intussusception [13, 22, 47, 49, 51]. Lung cancer manifesting as colonic metastasis is rare, and thus cited remotely in case reports. Broad interpretations based on such isolated events should be taken into consideration.

**Table 1:** Clinical presentations of patients with colonic metastasis, historically (1988–2016)

Case report	Cell type	Symptomatology
Azevedo et al. [30]	SqCC	Obstruction
Carroll et al. [20]	SqCC	Diarrhea
Cedres et al. [29]	SqCC	Abdominal pain
Franco et al. [31]	SqCC	Bloody stool
Gateley et al. [22]	SqCC	Hemorrhage
Gitt et al. [13]	SqCC	Perforation
Habesoglu et al. [19]	SqCC	Abdominal pain
Hirasaki et al. [17]	SqCC	Bloody stool
Lou et al. [32]	SqCC	Abdominal pain
Ma et al. [33]	SqCC	Abdominal pain
Rouhanimanesh et al. [34]	SqCC	Obstruction
Sakai et al. [25]	SqCC	Abdominal pain
Stinchcombe et al. [18]	SqCC	Incidental polyp
Wegener et al. [35]	SqCC	Bloody stool
Yuyuan Y. [26]	SqCC	Obstruction
Al-Tarakji et al. [39]	ADC	Encopresis
Ceretti et al. [41]	ADC	Obstruction
Hsing et al. [40]	ADC	Abdominal pain
Huang et al. [37]	ADC	Abdominal pain
Miyazaki et al. [38]	ADC	Abdominal pain
Ono et al. [52]	ADC	Abdominal pain
Pezzuto et al. [36]	ADC	Hyponatremia
Pozzato et al. [44]	ADC	Abdominal pain
Rossi et al. [10]	ADC, LCC	Polyp, bloody stool
Weng et al. [43]	ADC	Abdominal pain
Xue et al. [42]	ADC	Incidental polyp
Johnson et al. [48]	SCC	Bloody stool
Polak et al. [49]	SCC	Perforation
Yang et al. [5]	SCC	Bloody stool
Zhidong et al. [47]	SCC	Perforation
Goh et al. [51]	LCC	Hemorrhage
Chen et al. [53]	O	Abdominal pain
Lin et al. [54]	O	Intussusception
Bastos et al. [56]	U	Bloody stool
Myoteri et al. [55]	U	Incidental polyp

Particular case reports have been excluded from tabulation due to accessibility and non-English language barriers for symptomatology information only [21, 23, 27, 28, 45, 46, 50]

*SqCC* squamous cell carcinoma, *ADC* adenocarcinoma, *SCC* small cell carcinoma, *LCC* large cell carcinoma, *O* other: sarcomatoid or pleomorphic, *U* unknown

Initial diagnosis of colonic metastasis of lung carcinoma is challenging since its incidence has been reported sporadically. The phenomenon is being reported more frequently due to the recent higher rates of lung cancer in women, increased availability and utilization of endoscopic examinations, and advancements in IHC staining [9]. Details regarding colonic metastasis in terms of typical symptomatology remain sparse. Colonic metastasis of lung carcinoma can present as an incidental polyp, with bloody stool, or by significant bowel obstruction, such as with our patient.

Histological examination, in correlation with clinical findings, remains the gold standard for diagnosis. IHC stains such as TTF-1, CDX2, CK7, and CK20 help distinguish metastatic lung carcinoma from primary colonic cancer [10, 25]. The immunostaining profile of our patient (strongly positive CK7 and positive TTF-1 with negative CDX2/CK20) supported that her rectosigmoid tumor causing near-total rectal occlusion was metastatic adenocarcinoma of lung origin, rather than primary colorectal adenocarcinoma [57–59].

Early detection could be expedited by fecal occult blood testing [56]. This test is generally fast and inexpensive. As a result, stool testing is sufficient in terms of early evaluation and workup, especially in patients with abdominal symptoms and a known history of cancer [17]. PET-CT scans can diagnose asymptomatic colonic metastasis from lung carcinoma [5, 8, 18]. In contrast to conventional CT and endoscopy, PET-CT can determine if an intestinal neoplasm is of primary or secondary tumor origins. However, it is unable to establish an intestinal tumor’s specific histopathologic cell type.

Average survivability of patients with primary lung carcinoma, from the time of diagnosis of colonic metastasis to death, varies widely. Moreover, small and large bowel metastasis outcome data are often aggregated. The 5-year survival rate for stage IV metastatic NSCLC is approximately 10% [60]. Our patient initially received pembrolizumab before the discovery of colonic metastasis. Pembrolizumab is a novel and well-researched cancer immunotherapy most commonly used for tumors that are unresectable, recurrent, or metastatic [61]. Until recently, pembrolizumab has been recommended as a second-line agent. Combination chemotherapy with platinum-based pemetrexed and carboplatin is the first-line treatment for advanced NSCLC [62]. Trends are now focusing on tumor genotype-specific characteristics and in favor of earlier use of immunotherapeutic agents such as pembrolizumab. In a recent open-label phase III trial involving patients with advanced NSCLC, pembrolizumab was associated with significantly longer progression-free and overall survival [61–63]. Also, pembrolizumab was associated with fewer adverse events compared to platinum-based chemotherapy [61–63]. Before our patient’s initial presentation with symptomatic rectal occlusion, it was reported she did not tolerate pembrolizumab therapy well due to medication side effects.

All forms of intestinal metastasis of lung carcinoma are considered a late-stage complication of the disease. Average survival following the discovery of colonic metastasis to death has been reported to be approximately 2 months [5, 10, 56]. However, the range of survival after the diagnosis of colonic metastasis from primary lung carcinoma has been found to vary greatly [5, 7–14, 17–57]. Outcomes are based on chief complaints at the time of initial presentation and subsequent surgical intervention [5, 6, 11, 14, 15, 52, 53]. Perforation, obstruction, or hemorrhage have been associated with less favorable outcomes [6, 11, 19, 22, 48, 52, 53]. Early detection and surgical intervention have been postulated to improve survival [25]. Furthermore, longer survival times have been observed in patients that underwent palliative surgical resection of the metastatic site [8, 10–25], as with our patient.

## Conclusion

Colonic metastasis should be considered when patients have abdominal symptoms and a history of primary lung cancer. Expedited intestinal tract investigation should be done to allow for early detection and treatment. Findings can initially be subtle and isolated, such as a single polyp, bloody stool, or obstruction. Symptoms can be dismissed as a primary gastrointestinal process such as ulcers or colitis. Fecal occult blood testing, PET-CT scans, and endoscopy are clinically useful for establishing a diagnosis. However, histological examination confirms the diagnosis. Many previous case reports of this phenomenon present aggregate data from the small bowel and large bowel metastasis of lung carcinoma. More reports on colonic metastasis of lung carcinoma are required to clarify clinical features and outcomes. Ultimately, early detection and surgical intervention have been thought to improve survival.

## Abbreviations

CD45: Leukocyte common antigen
CDX2: Caudal type homeobox 2
CK20: Cytokeratin 20
CK5/6: Cytokeratin 5 or 6
CK7: Cytokeratin 7
CT: Computerized tomography
ER: Estrogen receptor
GCDFP-15: Gross cystic disease fluid protein 15
H&E: Hemotoxylin and eosin
IHC: Immunohistochemical
MART-1: Melanoma antigen recognized by T cells
Moc-31: Epithelial specific antigen/EpCAM
NCAM/CD56: Neural-cell adhesion molecule
NSCLC: Non-small cell lung carcinoma
PD-L1: Programmed death-ligand 1
PET-CT: Positron emission tomography–CT
SqCC: Squamous cell carcinoma
TTF-1: Thyroid transcription factor-1
